# Health occupations salary outcomes: intersections of student race, gender, and first-generation status

**DOI:** 10.1007/s10459-022-10154-2

**Published:** 2022-08-18

**Authors:** Peggy Gesing, Mohan D. Pant, Amanda K. Burbage

**Affiliations:** 1 Medical and Health Professions Education Program, Eastern Virginia Medical School, P.O. Box 1980, Norfolk, VA 23501 USA; 2 Master of Public Health, Eastern Virginia Medical School, Norfolk, USA

**Keywords:** Intersectionality, Critquant, Healthcare professionals, Healthcare education, First-generation students, NSCG

## Abstract

Greater diversity in the healthcare workforce has been identified as a critical need in serving an increasingly diverse population. Higher education institutions have been tasked with increasing the number of underrepresented students in the health occupations pipeline to better align with the demographics of the general population and meet the need for a diverse health occupations workforce. This study used the National Science Foundation’s National Survey of College Graduates dataset to capture data across time, examining the intersectionality of race, gender, and first-generation status on the salary outcomes of students who earn degrees related to health occupations. Results indicate that the intersecting identities of students who earn a bachelor’s degree or higher in the health professions impact salary outcomes. Results of this study have implications for higher education policies that can impact increased diversity in the health occupations workforce pipeline.

## Introduction

Disparities in healthcare and related health outcomes are known to exist in the population with lower-income racial minority groups lacking access to food, housing, jobs, health services, and education (Centers for Disease Control, n.d.; Jackson & Gracia, 2014; Manuel, 2018; World Health Organization [WHO], n.d.). These social determinants of health (SDH) are non-medical factors including conditions in which people are born and live, and the policies and systems that shape living conditions (WHO, n.d.). The SDH exist within and between countries with those in lower socioeconomic positions experiencing worse health and more illness. Understanding these, and other SDH, is imperative for health occupations educators working with an increasingly diverse provider population to deliver culturally competent care unconstrained by language and other barriers (Gilchrist & Rector, 2007; WHO, 2008). One strategy for reducing disparity and improving health outcomes for all populations is to increase diversity in the health occupations workforce to better align with the demographics of the general population (Bouye et al., 2016; Jackson & Gracia, 2014; Roberts et al., 2014).

Higher education institutions have made recruitment and retention of diverse student populations a priority in an effort to improve access to education for underrepresented students and to increase diversity in the STEM workforce (Gumport, 2016; Letizia, 2017). These diverse student populations include underrepresented students or students who are low-income racial and ethnic populations underrepresented in the medical professions relative to their numbers in the general population (Association of American Medical Colleges, 2004), and/or first-generation students whose parents or guardians never completed a four-year post-secondary degree (Ishitani, 2006; Spradlin et al., 2010). First-generation and underrepresented students face multiple barriers to higher education including financial and cultural barriers (Brigman, 2016; Demetriou, 2014; Dennis et al., 2005). These barriers are compounded for students pursuing careers in health occupations, with many health-related degrees requiring graduate-level credentials (Funk et al., 2018).

While studies have been conducted to gain an understanding of diverse student recruitment and retention trends, little research exists exploring the intersectionality of race, gender, and first-generation status on the salary outcomes of students graduating with degrees in health occupations. Intersectionality considers how an individual's different identities (e.g., gender, race, socioeconomic status, sexual orientation) accumulate to impact their lived experiences (Bi et al., 2020; Crenshaw, 1989) and highlights how the multiple components of identity must be considered when exploring social constructs (Crenshaw, 1991). The lack of insight into the impacts of intersectionality on salary outcomes has implications for attempts to increase and retain diversity in the health occupations workforce. This study explores salary outcomes of students who studied in health occupations in the U.S., analyzing data from the National Survey of College Graduates (NSCG) (U.S. Census Bureau, n.d.). Exploring NSCG data through an intersectional lens provides an opportunity to use a large, dataset to identify salary outcomes that go beyond one dimension of identity such as gender, race, or nationality.

The NSCG is a biennial survey that provides data on U.S. college graduates with a focus on students who go into the science and engineering workforce. This study examined the salary outcomes of students who graduated with health-related degrees who are working in health occupations and examined the intersectionality of outcomes by race, gender, and first-generation status. By examining intersectionality, we considered how students’ identities intersect with social structures to impact salary outcomes (American Psychological Association, 2017).

## Background

Intersectionality acknowledges that individuals’ multiple identities can multiply the impacts of discrimination (Crenshaw, 1989). Discrimination is often thought of as unidirectional, using single categories with considerations of gender focusing on discrimination of White women while considerations of race focus on Black men. The tendency to treat race and gender as mutually exclusive categories is problematic because this single-dimension view fails to consider the multiplied impacts of discrimination. Structural intersectionality acknowledges that multiple forms of structural oppression including racism, classism, and sexism impact those with intersecting identities within legal, financial, and healthcare systems (Crenshaw, 1991); however, the impact of this oppression is diffused when considered through a unidirectional lens (Haynes et al., 2020). Using an intersectional lens to explore human experiences at a societal and a healthcare system level can lead to greater visibility of marginalized groups in healthcare and health education (Muntinga et al., 2016).

## Intersectionality of students in health professions

Health professions educators have been called upon to use an intersectional approach to understand their students’ context, goals, and needs (Karani et al., 2017). Investigation of student outcomes through intersectional lenses have revealed differences in career pursuit (Charleston et al., 2014), awareness of microaggressions (Proctor et al., 2018), and ability to address complex intersectional clinical issues (Brinkman & Donohue, 2020). Espousing intersectionality in health professions education enables graduates and practitioners to embrace responsibilities, rectify imbalances, celebrate visibility, and engage diverse stakeholders in patient care environments (Eckstrand et al., 2016). Ultimately, understanding intersectional patterns among health professionals provides a basis upon which the reduction of health disparity improves public health through securing representation within the health system (Keshet et al., 2015).

## First-generation status

The intersection of race and socioeconomic status (SES) often impacts first-generation college students or those who are the first in their families to graduate from a post-secondary institution (Ishitani, 2006; Spradlin et al., 2010) making investigation of their intersectional experiences complex (Engle & Tinto, 2008; Haynes et al., 2020). First-generation students are more likely to be Black or Hispanic and therefore are often connected in research examining SES and underrepresentation (Chen, 2005, 2013). They share multiple characteristics including a lack of access to the income and college-going knowledge that non-first-generation students have (Chen, 2005; Lohfink & Paulsen, 2005; Luzeckyj et al., 2011). This leads to similar performance patterns that often negatively impact degree completion (Cahalan & Perna, 2015; Chen, 2005).

First-generation students tend to begin enrollment in community colleges and explore career options in vocational or technical fields where they can complete a degree or credential and join the workforce. This is compared to students whose parents have a bachelor’s degree or higher who are more likely to choose STEM or social sciences degrees that require a minimum of a four-year degree (Chen, 2005). However, first-generation students who explore career options in science at the bachelor’s degree level often focus primarily on medical-related careers and are influenced by potential compensation, alignment of career choice with personal interest, and ability to return home and contribute to their community (Dewsbury et al., 2019). First-generation students who do earn a bachelor’s degree have a harder time getting jobs, and when they do, they are often underemployed and earn lower wages than non-first-generation graduates (NASPA, 2019). In addition, they are less likely to pursue graduate education which is of particular concern in the health occupations where higher salary career options require post-baccalaureate education (Carlton, 2015; Funk et al., 2018). First-generation students who do pursue medical school report financial concerns, lack of health professionals in their network, lower levels of self-care, and environmental quality of life as barriers to education attainment (Brosnan et al., 2016; Mason et al., 2018).

This study examines salary outcomes of students who graduated with a health-related degree and were working in a health occupation at the time of the NSCG survey exploring how their intersectional identities impact salary outcomes. Salary was explored because it has been linked to health occupations staff retention but is known to vary based on job type (Gilles et al., 2014; Lu et al., 2019; Nemmaniwar & Deshpande, 2016). While various marginalized groups are differently impacted with inequity identification and outcomes, there is little research addressing these concerns for health care providers with intersectional identities (Silver et al., 2019).

## Framework

**Fig. 1:**
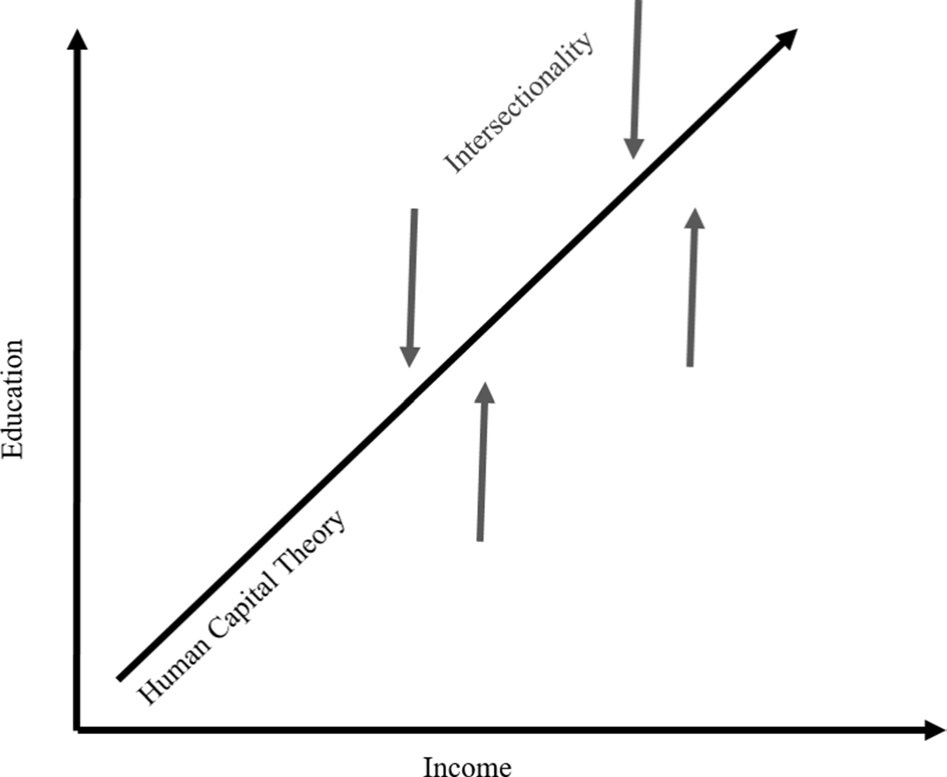
Human capital theory and critical quantitative intersectional framework

This study combines human capital theory framework and a critical quantitative intersectional approach to gain an understanding of how demographic variables intersect to impact career outcomes (Fig. 1). Human capital theory relates earnings to education and experience and suggests that individuals become more productive by investing in education, which improves career outcomes (Becker, 1992; Paulsen, 2001). Critical scholars centralize the experiences of marginalized groups; however, much of critical research uses qualitative methods to explore meaning and connection (Haynes et al., 2020; Jang, 2018). Scholars have begun to apply critical epistemologies to quantitative methods to focus on the systemic impact of marginalizing groups and challenging the assumed neutrality of numbers (Garcia et al., 2018; Gilborn et al., 2018). By combining human capital theory framework with a critical quantitative intersectional approach, researchers embrace the premise that diverse social categorizations mutually construct and intersect with systems of power (Jang, 2018).

This study contributes to research on higher education recruitment and retention and health occupations workforce development by exploring the impacts of the intersections of race, gender, and first-generation status on the salary outcomes of students with health-related degrees. The study’s outcomes have potential impacts on efforts to increase diversity in the health occupations workforce and has implications for higher education admissions and enrollment management policy.

## Methodology

Using NSCG data, this study examined the intersectionality of race, gender, and first-generation status on salary outcomes of students who studied in health occupations. The NSCG is a biennial survey providing data on U.S. college graduates and includes individuals living in the U.S. during the survey period who have at least a bachelor’s degree and are under the age of 76. This study used data collected via the 2015, 2017, and 2019 NSCG (U.S. Census Bureau, n.d.) to examine the salary outcomes of participants who graduated with health occupations degrees and were working in health occupations, comparing outcomes over time while examining the intersectionality of outcomes by race, gender, and first-generation status. Because this study utilized public-use NSCG data, demographic variables are limited by the instrument items. Terms used to refer to gender, race, and first-generation status are derived from the survey variables (Table 1).

**Table 1:** List of Variables

Variable group	Variable Code	Item Responses
Annual salary	SALARY	Numeric entry
Hours worked per week	HRSWK	< 35 h/wk > 35 h/wk
Job Category	N20CPR (N30CPR for 2019 data)	Biological/Life Scientists: Medical scientistsHealth Occupations: Diagnosing/treating practitioners, registered nurses, pharmacists, dieticians, therapists, physician assistants, nurse practitioners, psychologists, health technologists and technicians, other health occupations Managers, Other: Medical and health services manager
Most Recent Degree-Type	MRDG	1 Bachelor’s degree 2 Master’s degree 3 Doctorate 4 Other professional degree (e.g. JD, LLB, MD, DDS, DVM)
Most Recent Degree-Year	MRYR	Numeric entry *2005–2015
Most Recent Degree-Field of Study	NMRMED (N2MRMED for 2019 Data)	Coded entry list
First-Generation: Parent or Guardian Education	EDMOM EDDAD	1 Less than high school completed 2 High school diploma or equivalent 3 Some college, vocational, or trade school (including 2-year degrees) 4 Bachelor’s degree 5 Master’s degree 6 Professional degree (e.g. JD, LLB, MD, DDS, DVM) 7 Doctorate (e.g. PhD, DSc, EdD) 8 Not applicable
Race Hispanic	HISPANIC	0 No, not of Hispanic, Latino, or Spanish origin 1 Yes, Mexican, Mexican American, or Chicano 2 Yes, Puerto Rican 3 Yes, Cuban 4 Yes, another Hispanic, Latino or Spanish origin
Race	NATIVE	American Indian or Alaska Native
Race	PACIFIC	Native Hawaiian or other Pacific Islander
Race	ASIAN	Asian
Race	BLACK	Black or African American
Race	WHITE	White
Gender	GENDER	1 Male 2 Female

## Research questions

- RQ1: How do salary outcomes of students who graduate and work in health occupations vary by race, gender, and first-generation status?
- RQ2: What is the interaction of race, gender, first-generation status, and job type on the salary outcomes of students who graduate and work in health occupations?

## Data set

The sample for this study included 5375 participants (*n* = 2039 NSCG15, *n* = 1578 NSCG17, and *n* = 1758 NSCG19) who received a bachelor’s or higher degree in biological/life sciences or health and related sciences fields in the ten years before the survey and who indicated that their job category was health occupations or managers. Participants reported working at least 35 h per week while earning an annual salary of more than $10,000. Variable descriptions are based on the public-use NSCG15, NSCG17, and NSCG19 datasets (Table 1). Frequency distribution of the participants by intersectionality of the demographic categorical variables: race, gender, and first-generation status can be found in Table 2, and descriptive statistics are provided in Table 3. The variable that denotes first-generation status was derived using the condition: First-generation if both parents’ education level was below a bachelor’s degree.

**Table 2:** Three-way Frequency Distributions of Race, Gender, and First-Generation (SES)

		NSCG2015			NSCG2017			NSCG2019		
		First-Generation		Chi-sq(p-value)	First-Generation		Chi-sq(p-value)	First-Generation		Chi-sq(p-value)
Race	Gender	No	Yes	2.00(0.1577)	No	Yes	1.90(0.1678)	No	Yes	8.17(**0.0043**)
Asian	F	146	73		119	72		144	91	
	M	81	28		65	27		76	22	
ColumnTotal		227	101	34.04(**< 0.0001**)	184	99	10.14(**0.0015**)	220	113	22.50(< **0.0001**)
Non-Asian	F	727	589		575	411		646	458	
	M	278	109		207	95		228	83	
ColumnTotal		1005	698	0.03(0.8728)	782	506	0.96(0.3267)	874	541	0.001(0.9752)
Black	F	76	82		51	42		61	42	
	M	10	10		9	4		10	7	
ColumnTotal		86	92	35.74(**< 0.0001**)	60	46	11.27(**0.0008**)	71	49	32.71(< **0.0001**)
Non-Black	F	797	580		643	441		729	507	
	M	349	127		263	118		294	98	
ColumnTotal		1146	707	4.03(**0.0447**)	906	559	0.37(0.5451)	1023	605	8.16(**0.0043**)
Hispanic	F	75	76		48	50		55	58	
	M	33	17		15	12		26	8	
ColumnTotal		108	93	33.91(**< 0.0001**)	63	62	12.05(**0.0005**)	81	66	24.86(< **0.0001**)
Non-Hispanic	F	798	586		646	433		735	491	
	M	326	120		257	110		278	97	
ColumnTotal		1124	706	25.66(**< 0.0001**)	903	543	9.61(**0.0019**)	1013	588	17.24(< **0.0001**)
White	F	533	399		438	296		491	333	
	M	214	76		174	72		179	62	
ColumnTotal		747	475	12.54(**0.0004**)	612	368	3.28(0.0701)	670	395	14.38(**0.0001**)
Non-White	F	340	263		256	187		299	216	
	M	145	61		98	50		125	43	
ColumnTotal		485	324	3.55(0.0596)	354	237	0.19(0.6590)	424	259	0.35(0.5538)
AINHMR	F	43	32		38	23		39	25	
	M	21	6		9	7		13	6	
ColumnTotal		64	38	34.42(**< 0.0001**)	47	30	13.90(**0.0002**)	52	31	31.49(< **0.0001**)
Non-AINHMR	F	830	630		656	460		751	524	
	M	338	131		263	115		291	99	
ColumnTotal		1168	761		919	575	1.90(0.1678)	1042	623	8.17(**0.0043**)

Significance of *p* < 0.05 is indicated in bold

AINHM = American Indian/Alaska Native, Native Hawaiian/Other Pacific Islander, Multiple Race was created by combining "2: American Indian/Alaska Native, non-Hispanic ONLY","6: Non-Hispanic Native Hawaiian/Other Pacific Islander ONLY", and "7: Multiple Race, non-Hispanic" of RACETHM.

**Table 3:** Descriptive Statistics of Salary by Intersectionality of Race, Gender, and First-Generation

Race	Gender	FirstGen	NSCG2015			NSCG2017			NSCG2019		
			*M*	*SD*	*n*	*M*	*SD*	*n*	*M*	*SD*	*n*
Asian	Female	No	79,295	39,301	146	88,658	96,671	119	86,573	40,944	144
		Yes	88,688	46,691	73	102,433	117,704	72	100,451	73,427	91
	Male	No	84,189	111,682	81	112,970	98,577	65	119,037	101,181	76
		Yes	91,160	59,584	28	110,328	87,961	27	100,490	54,016	22
Black	Female	No	75,653	27,352	76	74,160	40,731	51	72,054	22,134	61
		Yes	78,048	30,141	82	79,933	28,446	42	77,566	28,113	42
	Male	No	77,520	30,009	10	82,889	31,446	9	69,030	33,780	10
		Yes	95,000	40,565	10	93,250	76,583	4	93,714	49,715	7
Hispanic	Female	No	77,487	33,828	75	88,667	70,237	48	87,878	52,268	55
		Yes	68,890	27,925	76	79,376	34,005	50	85,071	49,731	58
	Male	No	152,684	214,094	33	79,300	38,460	15	95,358	45,462	26
		Yes	84,442	69,486	17	71,829	23,327	12	77,625	29,154	8
White	Female	No	76,405	34,109	533	87,828	74,360	438	86,127	37,321	491
		Yes	83,788	87,823	399	85,859	42,184	296	88,080	50,830	333
	Male	No	109,350	141,448	214	128,556	156,168	174	126,753	124,662	179
		Yes	93,310	51,983	76	112,821	122,041	72	98,961	57,120	62
AINHMR	Female	No	66,475	22,391	43	69,463	29,889	38	69,543	21,947	39
		Yes	78,820	28,155	32	81,151	23,955	23	83,680	31,805	25
	Male	No	98,281	51,903	21	105,556	72,075	9	97,644	41,531	13
		Yes	74,167	11,232	6	68,286	30,170	7	67,676	12,255	6

Mean in thousands of annual salary dollars.

## Analysis and findings

Because salary data are usually non-normal, the log-transformation of annual salary was performed before answering research questions about salary outcomes. Data transformation was preferred over non-parametric statistical procedures centralized on the median because the null hypothesis for both research questions investigates differences of salary centralized on the mean. Multiple linear regression was used to answer RQ1, using white, non-first-generation, females as the comparison group. Full factorial design was used to answer RQ2.

Descriptive statistics (Table 3) indicate differences in salary outcomes based on the intersectionality of participants’ backgrounds. Overall, female participants reported lower salaries than male participants in all race and first-generation categories with Asian first-generation female participants earning the highest salary of all females and AINHMR^1^ non-first-generation female participants earning the lowest salary of all females. Among male participants, Hispanic non-first-generation male participants earned the highest salary, and AINHMR first-generation participants earned the lowest salary. First-generation Asian female, Black male, Black female, and AINHMR female participants in all samples (2015, 2017, & 2019) had higher mean salaries than non-first-generation participants of the same gender and race; while non-first-generation Hispanic female and Hispanic male, White male and AINHMR male participants had higher mean salaries in all samples. First-generation Asian male participants had higher mean salaries than non-first-generation Asian male participants in the 2015 sample and non-first-generation White female participants had higher mean salaries than first-generation White female participants in the 2017 sample.

RQ1: Do salary outcomes of students who graduate and work in health occupations vary by race, gender, and first-generation status? Multiple linear regression (Table 4) of the 2015 data revealed that no racial groups had a statistically lower salary outcome than the comparison group; however, gender was found to be a significant predictor, with male participants earning higher salary than female participants. There were differences in the 2017 and 2019 data with Black, Hispanic, and AINHMR, participants of both genders earning statistically lower salaries than White participants (*p* [< 0.0001.. 0.0625]). Male participants earned higher salaries than female participants (*p* =  < 0.0001). First-generation status was not a predictor of salary outcome in any group.

**Table 4:** Multiple Linear Regression Results for Research Question 1

	NSCG 2015				NSCG 2017				NSCG 2019			
Variable	β	SE	*t*	*P*	β	SE	*T*	*p*	β	SE	*T*	*p*
Intercept	4.85	0.01	652.57	** < 0.0001**	4.89	0.01	547.38	** < 0.0001**	4.90	0.01	628.30	** < 0.0001**
Asian	− 0.01	0.01	− 1.04	0.2980	− 0.01	0.01	− 0.61	0.5444	0.01	0.01	0.58	0.5599
Black	0.00	0.02	− 0.00	0.9983	− 0.05	0.02	− 2.19	**0.0290**	− 0.07	0.02	− 3.48	**0.0005**
Hispanic	− 0.02	0.02	− 1.20	0.2312	− 0.04	0.02	− 2.08	**0.0378**	− 0.03	0.02	− 1.86	**0.0625**
AINHMR	− 0.02	0.02	− 0.78	0.4349	− 0.06	0.03	− 2.15	**0.0319**	− 0.06	0.02	− 2.45	**0.0143**
Male	0.05	0.01	5.12	** < 0.0001**	0.07	0.01	5.07	** < 0.0001**	0.06	0.01	5.49	** < 0.0001**
First-Gen	0.01	0.01	0.96	0.3358	0.01	0.01	0.86	0.3897	0.00	0.01	0.21	0.8354

Significance of *p* < 0.05 is indicated in bold

*R*^2^ = 0.0137 (2015), *R*^2^ = 0.0248 (2017), and *R*^2^ = 0.0304 (2019). Dependent (response) variable = log_10_(Salary), the logarithm of Salary with base 10. For the variable race, the category White was used as a reference category. Thus, each of the four categories: Asian, Black, Hispanic, and AINHMR would be compared with White. The Male and First-Gen (abbreviated for First-Generation) were based on Gender (Male = 1, Female = 0) and First-Generation (Yes = 1, No = 0), respectively.

RQ2: What is the interaction of race, gender, first-generation status, and job type on the salary outcomes of students who graduate and work in health occupations? Full factorial design was used with log-transformed (base 10) salary as the dependent variable and race, gender, first-generation status, and job type as factors. Based on the model shown in Table 5, there was a significant main effect of job type on salary outcomes in all data samples (2015, 2017, and 2019); however, interaction effects varied. There was a significant interaction of race and job type (*p* = 0.0007) in the 2015 data sample, and a significant interaction of gender and job type (*p* = 0.0101) in the 2019 data sample. There were significant interactions of race and job type (*p* = 0.0495); race, gender and job type (*p* = 0.0375); and gender and first-generation status (*p* = 0.0309) in the 2017 data sample.

**Table 5:** Full Factorial Design with Log10Salary as Dependent Variable and Race, Gender, First-generation, and Job Type as Factors

	NSCG 2015					*NSCG 2017*					NSCG 2019				
Source	df	SS	MS	*F*	*p*	df	SS	MS	*F*	*p*	df	SS	MS	*F*	*p*
Race	4	0.19	0.05	1.22	0.2982	4	0.22	0.05	1.17	0.3218	4	0.22	0.06	1.50	0.1990
Gender	1	0.02	0.02	0.41	0.5202	1	0.00	0.00	0.00	0.9441	1	0.00	0.00	0.00	0.9768
Race*Gender	4	0.32	0.08	2.06	0.0839	4	0.04	0.01	0.23	0.9238	4	0.04	0.01	0.25	0.9077
First-Gen	1	0.02	0.02	0.63	0.4259	1	0.01	0.01	0.28	0.5955	1	0.05	0.05	1.37	0.2420
Race*First-Gen	4	0.23	0.06	1.46	0.2105	4	0.13	0.03	0.69	0.5955	4	0.08	0.02	0.52	0.7210
Gender*First-Gen	1	0.08	0.08	2.03	0.1547	1	0.21	0.21	4.67	**0.0309**	1	0.10	0.10	2.77	0.0962
Race*Gender*First-Gen	4	0.03	0.01	0.18	0.9498	4	0.14	0.04	0.78	0.5382	4	0.06	0.01	0.37	0.8290
Job-Type	3	1.30	0.43	11.29	** < 0.0001**	3	0.88	0.29	6.35	**0.0003**	3	0.68	0.23	6.07	**0.0004**
Race*Job-Type	12	1.32	0.11	2.85	**0.0007**	12	0.97	0.08	1.76	**0.0495**	12	0.44	0.04	0.98	0.4613
Gender*Job-Type	3	0.06	0.02	0.54	0.6566	3	0.31	0.10	2.21	0.0852	3	0.42	0.14	3.79	**0.0101**
Race*Gender*Job-Type	11	0.35	0.03	0.82	0.6248	10	0.89	0.09	1.93	**0.0375**	10	0.61	0.06	1.64	0.0895
First-Gen*Job-Type	3	0.14	0.05	1.24	0.2932	3	0.08	0.03	0.55	0.6467	3	0.07	0.02	0.64	0.5908
Race*First-Gen*Job-Type	12	0.44	0.04	0.95	0.4913	10	0.12	0.01	0.26	0.9896	12	0.21	0.02	0.47	0.9333
Gender*First-Gen*Job-Type	3	0.03	0.01	0.22	0.8799	3	0.06	0.02	0.45	0.7198	3	0.15	0.05	1.36	0.2534
Race*Gender*First-Gen*Job-Type	8	0.50	0.06	1.62	0.1141	7	0.20	0.03	0.63	0.7311	5	0.01	0.00	0.03	0.9996

Significance of *p* < 0.05 is indicated in bold

*R*^2^ = 0.105 (2015), *R*^2^ = 0.098 (2017), and *R*^2^ = 0.135 (2019) implying that 10.5%, 9.8% and 13.5% of variance in log(salary) is accounted for by the factors (Race, Gender, First-Gen, and Job-Type) and their interactions in the model.

## Discussion

The results of this study indicate differences in salary outcomes of students who graduate and work in health occupations by race, gender, and first-generation status. However, the differences vary between intersectional factors, illustrating the impact of multidimensional identities and affirming the use of human capital theory framework and a critical quantitative intersectional approach.

Empirical evidence shows that in many developed countries salaries of racial and ethnic minorities are on average, less than the White native majority (Longhi, 2020). This difference is also true of gender with international salary data showing that men out-earn women in each racial group (Kane et al., 2021; Organisation for Economic Co-operation and Development, n.d.; U.S. Bureau of Labor Statistics [USBLS] 2016, 2018, 2020). Female participants in this study in all racial groups reported lower salaries than males which aligns with international salary statistics. Although this study limited the sample to those working 35 or more hours per week, there are important reasons salary may vary which exacerbate the gender salary gap. Health professionals likely receive pay differentials up to 10% of overall pay under federal systems for working night shifts (OPM, n.d.) and time-and-a-half pay for overtime (U. S. Department of Labor, 2009); however, female health professionals report preferring day shifts (Stimpfel et al., 2019) and are less likely to work overtime (Anxo & Karlsson, 2019).

Quantitative investigations of intersectional identities are under-explored and not uniformly approached (Guan et al., 2021). Nevertheless, intersectionality has been linked with economic insecurity and perceived job insecurity where individuals with multiple marginalized identities experience hierarchies of disadvantage (Lavaysse et al, 2018; Maroto et al, 2019). In this study, the complexity of intersectional identities was not universally observed. Mean salary data compared by race indicated that Asian and White female participants earned more on average than female participants in other race groups, while Hispanic and White male participants earned more on average than male participants in other race groups. This finding is counter to U.S. Bureau of Labor Statistics data which indicate that although Hispanic males have the highest employment-population ratio, or proportion of the population that is employed, Black and Hispanic males working full-time earned less than White and Asian males (USBLS, 2016, 2018, 2020). This finding requires follow up when reviewing future NSCG data sets.

In the United States it is illegal to discriminate against an employee or job applicant on the basis of race, color, religion, sex, national origin, age, disability, or genetic information (U.S. Equal Employment Opportunity Commission, 2022), leading researchers to focus on these identities when studying intersectionality. While other identities may be considered in intersectional research, few studies include first-generation status. This study provides an additional perspective for how first-generation status interacts with students’ other identities to impact their salary outcomes. Because first-generation students are more likely to be Black or Hispanic their intersecting identities may impact monetary and non-monetary career outcomes. First-generation status may have an indirect impact on salary outcomes because first-generation college graduates are more likely than non-first-generation graduates to be under-employed, work for a non-profit, or work in government roles where salary is often lower (NASPA, 2019). These economic outcomes do not take into consideration the non-monetary benefits of a college degree or value that is placed on jobs committed to service and social justice (Pérez Huber et al., 2018). Educational attainment is known to impact salary outcomes with college graduates with advanced degree out earning those holding a bachelor’s degree or less (Longhi, 2020; USBLS, 2021); however, first-generation students are less likely to earn the advanced degrees required for increased health occupation salaries (Funk et al., 2018), therefore the misalignment of the findings of this study with labor data warrant further exploration.

Although first-generation status was not found to be a statistically significant predictor of salary outcome in this study, mean salary differences were identified with first-generation participants in several minority racial groups out earning non-first-generation participants. Asian, Black, and AINHMR female and Black male first-generation participants reported higher salaries than non-first-generation participants in the same racial and gender groups. This supports Manzoni and Streib’s (2019) findings that first-generation status does not contribute to the wage gap for female students in health majors, and instead, geographic location and labor market sector are the biggest contributors to the wage gap. Results exploring the intersection of race, gender, and first-generation status on salary by job type indicated a significant main effect of job type on salary outcomes in all data samples with varied interaction effects. Although the interactions of race and job type; gender and job type; race, gender, and job type; and gender and first-generation status were not consistent from sample to sample, the presence of these significant interactions should be examined in future datasets to identify possible trends. The impact of job type supports data which indicate that health care practitioner roles requiring an advanced degree like physician, and physician assistant are held primarily by White employees, while roles requiring less than a bachelor’s degree are held by a larger percentage of Black and Hispanic employees (National Center for Health Workforce Analysis, 2017; USBLS, 2016, 2018, 2020). As Dhamoon (2011) stated “there are no universal grounds on how to know which interactions should be studied” (p. 239); however, choice of interaction effect in the context of this study should be made explicit (Choo et al., 2010). Considering social justice related to diversity in health professions student populations while working within the boundaries of the NSCG secondary dataset, we utilized AAMC defined parameters for underrepresented students leading to the identification of race, gender, and first-generation status to explore interactions related to job type and salary outcomes.

Ultimately there are important distinctions between race, SES, and first-generation status, further nuanced by intersectional identities which may involve any, all, or other predefined categories. For instance, Nguyen and Nguyen (2018) suggested investigations in first-generation experiences are best operationalized within their relationships to other identities. However, the predefined nature of the secondary dataset and fixed social categories of variables limited the opportunity to parse out these potentially important distinctions (Gillbourn et al., 2018). Other limitations to consider include the timeframe in which the data were collected. Because data in each sample included participants who graduated ten years prior to the survey year, impacts of the great recession that occurred from 2007–2009 must be taken into consideration. In addition, comparison of these data sets to the 2021 and 2023 NSCG data sets will require consideration of the financial crisis, focus on racial disparities, and impacts on healthcare professions brought on by the Coronavirus pandemic. The NSCG data is collected from U.S. college graduates who were residing in the U.S. at the time of the survey. Results should be considered within the U.S. context while considering implications for international health professions workforce trends.

This study identifies areas requiring further research related to the salary outcomes of students entering the health occupations workforce. Although the analysis indicated significant differences by job type, there is a need to disaggregate the data by healthcare degree earned, and healthcare job category. There is also a need to decentralize White males as the normative quantitative measure, and although this study used White females as the comparison group, another race category may be better suited as the central comparison through an intersectional lens. The disparities in income by race, gender, and first-generation status are factors that can impact students’ investment in graduate education and career decisions as they explore potential degree and career fields. Continuing to analyze NSCG biennial data can provide additional insights into trends in salary outcomes; however, including comparisons to international datasets will allow researchers to examine intersecting identities in a global context. Finally, qualitative research could provide a deeper understanding of the stories behind the data, illustrating how intersecting identities impact career outcomes by giving voice to the intersecting identities studied.

This study begins to address questions about the impact of students’ intersecting identities on their career outcomes with a focus on healthcare occupations. In doing so, we extend the research related to increasing diversity in the health occupations pipeline. Higher education and healthcare institutions committed to building a diverse health occupations workforce can benefit from these findings by considering the intersecting identities of health care professionals, striving for salary parity in the health occupations workforce.

## Footnotes

1 The variable AINHMR was created due to the small number of participants in the American Indian/Alaska Native, Native Hawaiian/Other Pacific Islander, Multiple Race categories.
